# Surgical Cases

**Published:** 1865-10

**Authors:** E. Andrews

**Affiliations:** Prof. of Surgery in Chicago Medical College


### ARTICLE XXXIV.

### SURGICAL CASES.

By E. ANDREWS, M.D., Prof. of Surgery in Chicago Medical College.

Case I.—*Varicose Ulcer treated by Hypodermic Injection.* The cases of varicose veins treated by injection, which I published in the Examiner two months since, have had the effect to elicit considerable inquiry on the subject, as shown by numerous letters which I have received. The following additional one may be of first interest.

Michael Kenny was admitted to Mercy Hospital, six months ago, with a varicose ulcer on the left leg, about an inch and a quarter in diameter. The patient was a hearty, well-fed Irishman, inclining to corpulence. The ulcer was indolent, with thickened edges and indurated base, the floor of it feeling hard, and having little sensitiveness. The veins around it were varicose, some of them extending beneath the ulcer, and being marked by compressible streaks in the hard floor. This man was treated by every ordinary method which promised success, in vain. He had sulphur and muriate of ammonia internally, and nit. silver, sul. copper, scarifications, adhesive straps, olive oil and turpentine, chloride of zinc, muriate of ammonia, and, finally, caustic potash, externally. Nothing had any more than a slight effect, and, at the end of six months, he was scarcely changed in his condition. The patient had resisted all advice to submit to hypodermic injection, but at length concluded to try it. I injected about three drops of tinct. ferr. mur. into each of about five varicose places, two of which were in the veins under the floor of the ulcer. Inflammation, to some extent, followed. The patient was treated by giving tinct. iron freely internally, and carefully supplied with a constant current of fresh air, to prevent aplastic phlebitis, and with an astringent lotion externally. A small abscess formed at one of the points of injection, which was lanced, and the whole affected tract was red and inflamed. The ulcer underwent no remarkable change until the abscess discharged, and the inflammation began to abate. The edges of the ulcer then commenced rapidly to cicatrize, and at the present time is nearly healed.

CASE II.—*Hydrocele.* This disease is commonly treated either by the seton, or by injections of tinct. iodine. In both these methods we are usually successful at the first operation. This and the following case, however, are introduced to show that both operations occasionally require repetition before they are completely successful.

A. B., a young man, had a hydrocele of two years' standing. It had been palliated occasionally by tapping—of course, without permanent advantage. A timid physician determined to inject the iodine, but fearing inflammation, he only used a very weak solution. The result was partly favorable, obliterating the upper part of the sac, but not the lower. He then sent the patient to me. I tapped the remainder of the sack, and threw in the tinct. of iodine in full strength. After keeping it there a few moments, it was allowed to run out of the pipe, as usual, and the tube withdrawn. This resulted in a moderate inflammation, and an obliteration of still more of the sack. When the inflammation subsided, there still remained a small hydrocele at the lower part of the tunica vaginalis. This I obliterated by a third injection, which completed the cure.

CASE III.—*Hydrocele requiring repetition of the seton.* A

H., a German, fifty-two years of age, and of good constitution, had a hydrocele for several years. The walls of the sac were so thick that no light from a candle could be passed through them; hence the ordinary translucency could not be detected, but an exploring trochar proved the nature of the contents. I determined to treat it by seton. A tape was therefore passed through it from below upwards, along which the fluid drained away. In four days a smart inflammation was set up, which caused the tumor to be swollen to its original size. The seton was then removed, and the inflammation gradually subsided, when the lower part of the sac was found not to be obliterated, and to be filling again with fluid. I inserted another seton, which I left in about a week, which completed the cure.

On the whole, I find that there is very little ground of choice between these operations, both being usually successful, but each of them, in a few cases, requiring repetition.

Case IV.—*Anchylosis of the knee.* A large number of anchylosed knees are grossly neglected by the profession. Many practitioners seem to be unaware of the fact, that nearly all the stiff knees which hobble around with the limb fixed at a right angle, and with the foot unable to reach the ground, can be straightened without risk, and often without even cutting the tendons.

C. Y., an Indian boy, in Mackinaw, was sent to me by a gentleman who took pity on him as a cripple. He had cut his knee with an hatchet, resulting in suppuration of the joint, which subsequently healed with anchylosis, and the knee fixed at a right angle on the thigh. The flexor muscles were also contracted. I cut both hamstrings, and applied a jointed splint, which was gradually extended by a screw brace. In six weeks the limb was completely straightened, and he was sent home with instructions to wear a straight splint until there was no tendency to a return of the flexure. The patient not remaining long enough, I could do nothing towards restoring motion, but still the limb will be strong, and enable him to go without crutches.

Case V.—C. F., a girl twelve years old, had caries of the right knee, with absorption of the head of the tibia, and fistulous openings into the joints. After nearly recovering from the inflammation, and after the fistulas were nearly healed, she was brought to me with the knee anchylosed, bent at a right angle, and the tibia slightly displaced backwards and outwards. An extension splint was applied without cutting the tendons, and the limb was straightened in about eight weeks.

CASE VI.—S. H., a boy aged thirteen years. He had caries of the knee, with destruction of part of the head of the tibia, some years ago, which gradually healed, leaving the knee deformed exactly like Case V., but worse. Fistulas all healed, and the knee bent at about a right angle. Applied an extension splint, which brought the foot to the ground in about ten weeks. He now walks about with a staff, keeping the limb in a straight position by means of a straight splint.

CASE VII.—A woman aged thirty. Has had an inflammation of the right knee without caries. It is bent nearly to a right angle, but preserves a slight mobility. I applied the extension splint, which has brought down the foot more rapidly than usual. As it now appears, it seems that two weeks application of the splint will completely straighten the limb. Exercise will probably restore a degree of mobility in this case.

In many of these cases it is not possible to restore the control of the extension muscles over the limb, on account of the patella being firmly glued to its position, so that it will not glide on the joint, and we have no effectual and safe way of dislodging it; still, if the foot is brought to the ground, and the knee remains stiff in a nearly straight position, it immediately becomes of use in walking. The patient is able to dispense with crutches, and move about again in something like a natural manner.

